# [Corrigendum] Inhibition of DNA-PK activity sensitizes A549 cells to X-ray irradiation by inducing the ATM-dependent DNA damage response

**DOI:** 10.3892/mmr.2026.13890

**Published:** 2026-04-27

**Authors:** Lina Yang, Xinrui Yang, Yiwei Tang, Defu Zhang, Lijie Zhu, Shengnan Wang, Bo Wang, Tao Ma

Mol Med Rep 17: 7545–7552, 2018; DOI: 10.3892/mmr.2018.8828

Following the publication of the above article, a concerned author drew to the authors’ attention that, in Fig. 1B on p. 7548, the first two lanes for the lower set of β-actin western blots were unexpectedly similar to the data shown for the ATM protein blots in the third and fourth lanes in the same figure part. In addition, the upper set of β-actin western blots featured in Fig. 1B were also strikingly similar to the blots shown for the Bax protein in Fig. 2 on p. 6549. Moreover, an independent analysis of the data in this paper undertaken by the Editorial Office also revealed that the TAp73 and GRAMD protein blots shown in Fig. 2 were very similar.

After having re-assessed these figures, the authors have submitted corrected versions of Figs. 1 and 2, as shown on the next page, featuring revised data for the upper and lower sets of β-actin blots in Fig. 1, and the TAp73 blots and certain of the β-actin blots in Fig. 2. Note that these errors did not affect the results or the main conclusions reported in the study. All the authors approve of the publication of this corrigendum, and are grateful to the Editor of *Molecular Medicine Reports* for allowing them the opportunity to publish this. The authors regret their oversight in allowing these errors to be included in the paper, and apologize to the readership for any inconvenience caused.

## Figures and Tables

**Figure 1.**
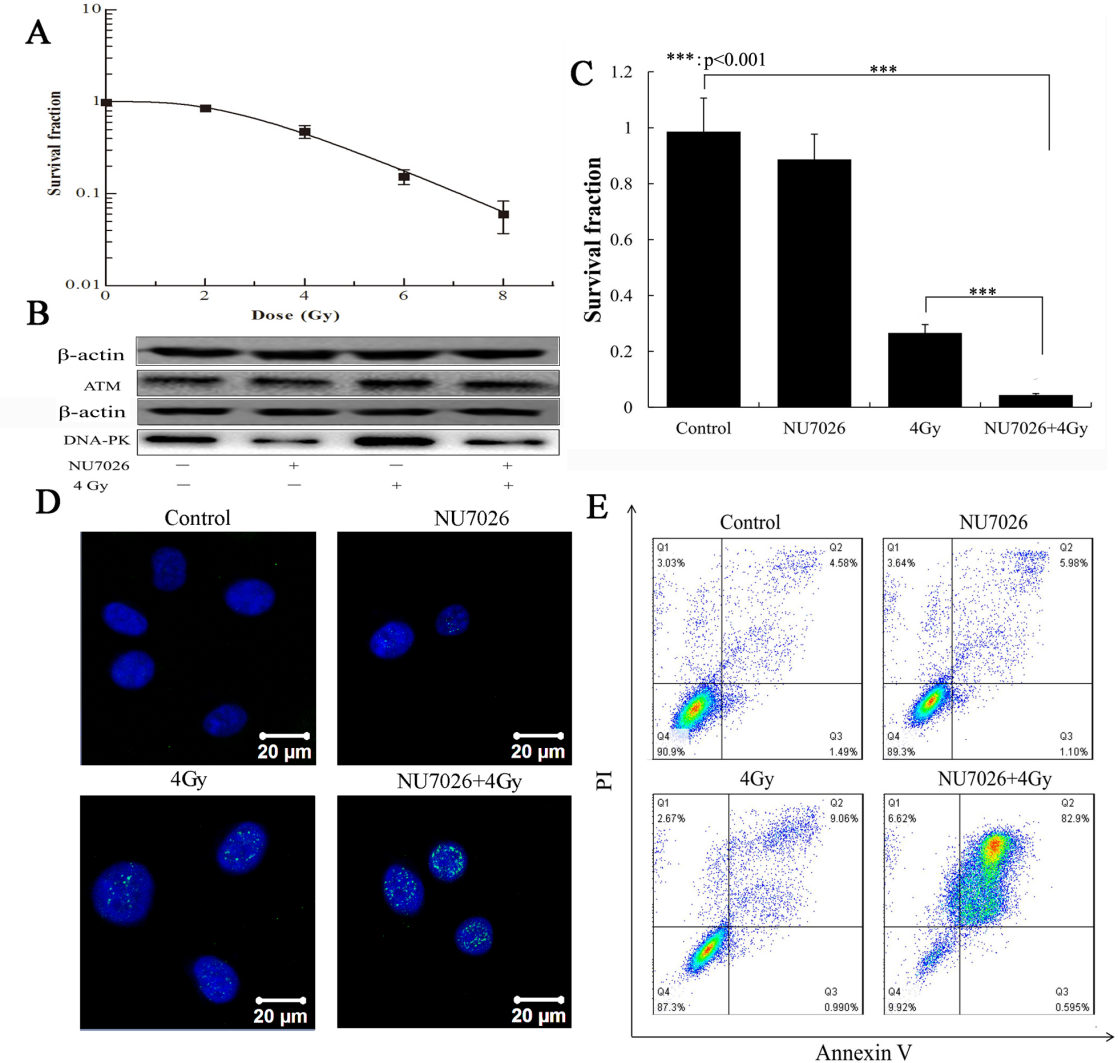
Effects of NU7026 and X-rays on growth, DNA damage and apoptosis in A549 cells. (A) The survival fraction was calculated after 0, 2, 4, 6 and 8 Gy X-ray irradiation. (B) Protein levels of DNA-PK and ATM were determined by western blotting 24 h post-treatment, following treatment with no-irradiation, 10 µM NU7026, 4 Gy X-rays and 10 µM NU7026+4 Gy X-rays. (C) The survival fraction was calculated after 10 µM NU7026 and 4 Gy X-ray irradiation. ***P<0.001. (D) γH2AX foci were imaged and detected by immunofluorescence 0.5 h post-irradiation. (E) Apoptosis was analyzed by flow cytometry after Annexin V/PI staining 24 h post-irradiation.

**Figure 2.**
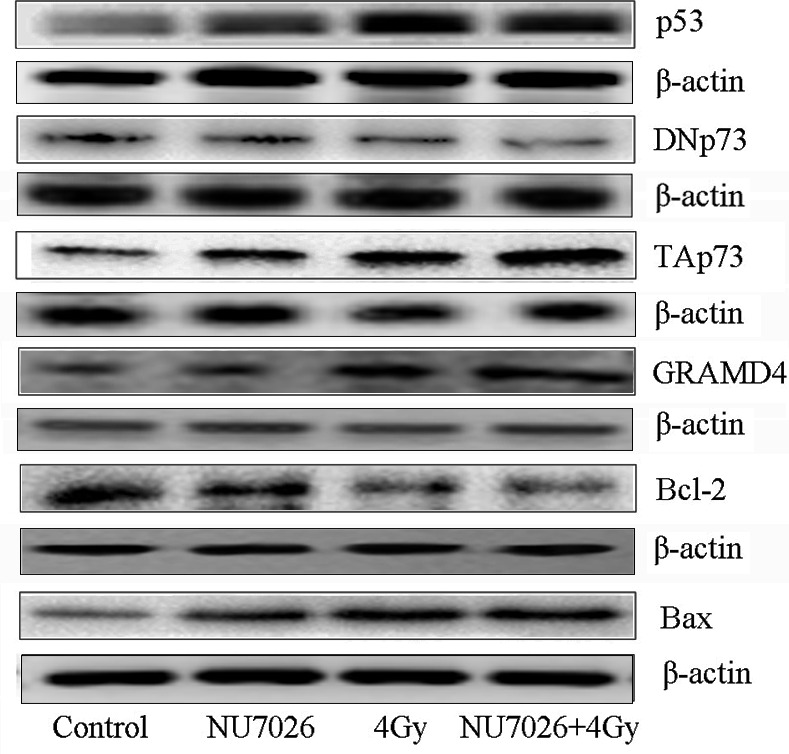
Effects of NU7026 and X-rays on expression of apoptosis-related proteins in A549 cells. The protein levels of p53, DNp73, TAp73, GRAMD4, Bcl-2 and Bax were determined by western blotting 24 h post-irradiation, following treatment with 10 µM NU7026 for 30 min and 4 Gy X-rays. ***P<0.001 vs. the control group, ^#^P<0.05 and ^##^P<0.01 vs. the 4 Gy group.

